# Blindness and visual impairment and their causes in India: Results of a nationally representative survey

**DOI:** 10.1371/journal.pone.0271736

**Published:** 2022-07-21

**Authors:** Praveen Vashist, Suraj Singh Senjam, Vivek Gupta, Noopur Gupta, B. R. Shamanna, Meenakshi Wadhwani, Pallavi Shukla, Souvik Manna, Saumya Yadav, Amit Bharadwaj

**Affiliations:** 1 Community Ophthalmology, Dr. R P Centre, AIIMS, New Delhi, India; 2 Ophthalmology, Dr. R P Centre, AIIMS, New Delhi, India; 3 School of Medical Sciences, University of Hyderabad, Hyderabad, Telangana; 4 Chacha Nehru Bal Chikitsalaya, New Delhi, India; 5 Institute Rotary Cancer Hospital, AIIMS, New Delhi, India; 6 AIIMS, New Delhi, India; Saarland University, GERMANY

## Abstract

**Introduction:**

Avoidable blindness is a significant public health problem in India. Nationally representative RAAB surveys (Rapid Assessment of Avoidable Blindness) are being conducted periodically in the country to know the current status of blindness in the country. The current study describes the findings from the RAAB survey conducted during 2015–19 in India.

**Methodology:**

A cross-sectional, population-based survey was conducted across the entire country among persons aged 50 years and above using RAAB version 6 methodology. Presenting and pinhole visual acuity was recorded followed by lens examination using a torchlight. In order to estimate the prevalence of blindness and visual impairment in overall population in India, district weights were assigned to each of the 31 surveyed districts and the prevalence was standardized using the RAAB software.

**Results:**

The overall weighted, age-gender standardized, prevalence of blindness (presenting visual acuity <3/60 in better eye) in population aged ≥50 years was 1.99% (95% CI 1.94%, 2.13%) and of visual impairment (VI) (presenting visual acuity <6/12 in better eye) was 26.68% (95% CI 26.57–27.17%). On multivariate analysis, adjusted odds ratio showed that blindness was associated with age ≥ 80 years (OR = 20.3, 95% CI: 15.6–26.4) and being illiterate (OR = 5.6, 95% CI: 3.6–8.9). Blindness was not found to be significantly associated with either gender or locality.

**Conclusion:**

The results of the survey demonstrate that currently more than one fourth of persons aged 50 years and above are visually impaired (PVA<6/12 in better eye) in India. The prevalence of blindness among them is 1.99%, and older age and illiteracy are significantly associated with blindness. Major causes of blindness included cataract (66.2%), corneal opacity (CO) (8.2%), cataract surgical complications (7.2%), posterior segment disorders (5.9%) and glaucoma (5.5%). The proportion of blindness and visual impairment that is due to avoidable causes include 92.9% and 97.4% respectively.

## Introduction

Globally, at least 2.2 billion people have a vision impairment, and of these, at least 1 billion people have a vision impairment that could have been prevented or is yet to be addressed [1]. The definitions of blindness have evolved over the years internationally and in India [2]. A contemporary blindness study from Central Europe defined blindness as visual acuity ≤ 1/60 without visual field defects, as visual acuity ≤ 2/60 with quadrantanopsia, as visual acuity ≤ 4/60 with hemianopsia, or as visual acuity 6/60 with tunnel vision [3]. Similarly, it defined visual impairment as visual acuity ≤ 3/60 without visual field defects, as visual acuity 6/60 with quadrantanopsia, as visual acuity 6/20 with hemianopsia, or as visual acuity ≤ 6/6 with tunnel vision. The WHO definition of blindness, used in the current study, is slightly less strict than the definition used in Europe and defines a presenting visual acuity (PVA) of 3/60 or worse as indicating blindness; and PVA <6/12 as visual impairment [4]. Blindness is a significant public health problem globally with a disproportionately large prevalence in low and middle income countries (LMICs) [5]. Various efforts have been made at the global level to address this situation. In 2013, the World Health Assembly launched Global Action Plan (WHA GAP) with a target of achieving 25% reduction in prevalence of avoidable visual impairment by 2019 compared to the baseline levels of 2010 [6]. Recent reports by Vision Loss Expert Group (VLEG) of the Global Burden of Disease (GBD) Study show that there are approximately 33.6 million blind people and 206 million people with moderate to severe visual impairment globally [7]. Although, there is a decline in the prevalence of avoidable visual impairment, especially in adults aged 50 years and above, the WHA GAP target has not been met [7].

India has implemented a series of effective measures in its ongoing National Program for Control of Blindness and Visual Impairment (NPCB&VI) to combat the situation, and it has resulted in a significant decline in prevalence of blindness over the past few decades [8, 9]. Periodic updates in current estimates and causes of blindness and visual impairment are essential to assess the effectiveness of ongoing public health policies and provide a sound basis for development of future strategies and their effective implementation. The Rapid Assessment of Avoidable Blindness (RAAB) has been developed as a simple and rapid survey methodology that can provide data on the prevalence and causes of blindness [10]. RAAB was developed by Hans Limburg working with the International Centre for Eye Health, first as the Rapid Assessment of Cataract Surgical Services in 1997, and then updated and modified to create RAAB in 2004 [11–13].

Currently, various methods are in use for rapid assessment of visual impairment, but largely these methods are used for older adults (50 years and above), including RAAB [14]. The current study was a nationwide RAAB survey conducted in randomly selected 31 districts of India with the assistance of NPCB&VI, India. The objective was to document the status of blindness and provide evidence-based data on the prevalence and determinants of blindness and visual impairment in population aged ≥50 years in the country. The findings of the survey will also help in monitoring the trend of blindness over time for programmatic purpose.

## Methodology

This was a cross-sectional, population-based survey conducted across the entire country during 2015–19 among population aged 50 years and above. The survey protocol was approved by the Institute Ethics Committee, All India Institute of Medical Sciences, New Delhi, India. Approvals from state and district health authorities were obtained before initiating survey activities and the study was conducted as per the tenets of the Declaration of Helsinki. Written informed consent was obtained from every participant and any participant requiring ophthalmic care was offered referral services to the nearest eye care centre.

The Rapid Assessment of Avoidable Blindness (RAAB) version 6 methodology was utilized, which used paper-based data collection and Snellen E optotypes for visual acuity. The RAAB subsequently launched version 7 in 2021 which facilitated greater use of mobile technologies and a greater emphasis on using the data for planning [15]. Eligible Subjects included population aged 50 years and above residing in the selected household for the past six months or more. “Residing in household” was defined as sharing meals from the same kitchen with other household members.

### Sample size and sampling

The survey was designed to be representative of the entire country. The sampling frame comprised of all the 631 districts of India as per the 2011 census and 31 districts were randomly selected using probability proportionate to size (PPS) systematic random sampling. Based on 3.6% estimated prevalence of blindness, 25% relative precision, 95% confidence interval, design effect of 1.6, and coverage of 90%, sample size for each district was calculated as 3000 [6]. Within each district, 50 clusters were again selected using PPS sampling. Within a selected cluster, compact segment sampling was utilized by the field team using draw of chits. Within the selected segment, households were selected from a random starting point, and a line listing was done, till the sample size of 60 per cluster was achieved. This survey also included a module to assess diabetic retinopathy (DR) in selected districts having a high prevalence of diabetes, the results of which are published already [16]. The 50 clusters included a representative proportion of rural areas (villages) and urban wards, based on the rural-urban distribution of the district population. An urban area, according to the Census definition, consists of [17]:

1. all statutory towns: All places with a municipality, corporation, Cantonment Board or notified town area committee, etc. so declared by state law.
2. Census towns: Places with a minimum population of 5000, at least 75 percent of male working population engaged in non- agricultural pursuits and a density of population of at least 400 persons per square km.

In addition, some areas falling in the vicinity of city or town are also considered as urban area if they are treated as the out growths (OGs) of the main urban unit. Such OGs are shown as urban agglomerations. As per the census definition, urban agglomeration is a continuous urban spread constituting a town and its adjoining urban outgrowths (OGs) or two or more physical contiguous town together and any adjoining urban out growths of such towns. All remaining areas which are not urban are classified as rural, and the basic unit for rural areas is the revenue village.

#### Clinical assessment

Clinical examination of study participants aged 50 years and above was performed as per RAAB version 6 methodology. Age, gender, and educational status of each participant were documented and presenting visual acuity (PVA) was assessed using Snellen `E’ optotypes sized 6/12, 6/18 and 6/60, starting with the largest size from a distance of 6 metres. Pinhole visual acuity was assessed in case the PVA was less than 6/12 in order to identify refractive errors. Assessment of anterior segment and lens status was done using a portable slit lamp and distant direct ophthalmoscopy. In case of vision loss with no apparent anterior segment cause, dilated fundus evaluation was done using direct and/or indirect ophthalmoscope. The ophthalmologist noted the principal cause of visual impairment for any eye if the presenting visual acuity was less than 6/12, and the principal cause for the participant was taken as that in the better eye. Only one diagnosis was marked for each eye and one diagnosis for the participant. In case there were two or more co-existing primary conditions in one eye, the more avoidable/treatable cause was marked as the principal cause.

Prior to initiation of the RAAB survey, the entire team was trained and certified in the RAAB methodology by an accredited RAAB trainer as per the survey protocol. Before undertaking the RAAB survey, inter-observer variation (IOV) test among optometrists was performed for presenting and pinhole vision examination in each eye. For ophthalmologists, IOV test was done to measure agreement on the assessment of lens status in each eye and cause of visual impairment in each eye and also in the participant. The findings of each ophthalmologist were compared with the findings of the most experienced ophthalmologist, the so-called ‘Gold Standard’. The RAAB package module was used to calculate the IOV, which was expressed in the Kappa coefficient. Any team scoring a Kappa of less than 0.60 was trained again before participation in the fieldwork. A standardized manual of operations was developed for the survey.

### Data analysis

The data was entered in RAAB-6 software package (50+ survey) on the same day that the survey was conducted. Transcription errors were minimized through dual-data entry and double data entry validation. The corrected dataset was analysed using inbuilt routines and validation checks of the RAAB6 software. Standardized definitions were used for classifying participants’ visual status and diagnosis. (Table 1) Data within each district was directly age-sex standardized by the in-built analysis routines of RAAB-6, using the age-sex distribution of the 2011 national census population. In order to calculate the district weights, the population that was aged 50 years and above in each of the sampled districts was divided by the total population that was aged 50 years and above in all 31 sampled districts. Subsequently, country-level estimates were obtained by multiplying the age-sex standardized prevalence with the respective district weights and taking the cumulative sum of all 31 districts. Calculations were done separately for male and female population, and also for combined population. The data was exported as comma separated files for univariate and multivariate analysis using Stata (StataCorp, College Station, Texas). For all point estimates, 95% confidence intervals were calculated, and the critical significance value was fixed as p value < 0.05.

**Table 1:** Definitions of vision impairment used in the National Blindness and VI Survey.

Category	Definition (based on presenting visual acuity of better eye with available correction)
Blindness	< 3/60
Severe visual impairment	< 6/60–3/60
Moderate visual impairment	< 6/18–6/60
Mild visual impairment	< 6/12–6/18
Moderate-severe visual impairment	<6/18–3/60
Visual impairment	< 6/12
Pinhole Blind	Best corrected vision <3/60 in better eye
Functional low vision	A person with impairment of visual functioning even after treatment and/or standard refractive correction, and a visual acuity of less than 6/18 to light perception, or a visual field of less than 10 degree from the point of fixation, but who uses, or is potentially able to use, vision for planning and/or execution of a task.

## Results

Overall, 93018 individuals (≥ 50 years of age) were enumerated from 31 districts, out of which 85135 completed all study procedures and ophthalmic assessment (participation rate 91.5%). A higher participation was observed in females (94.9%) in comparison to males (87.4%). Mean age of the study participants was 61.6 (±9.6) years (range, 50–99 years) and 57.3% of the participants were females. Mean age of males was 62.4 (±9.5) years (range, 50–99 years) and that of females was 61 (±9.5) years (range, 50–99 years). There was no significant difference between age and gender of the participants and non-participants. Overall, 66,304 (77.9%) participants resided in rural areas and 57.6% were illiterate (Table 2).

**Table 2:** Socio-demographic profile of study participants aged ≥50 years.

	Enumerated (N = 93018)			Examined (N = 85135)		
Age	Male (N = 41566)	Female (N = 51452)	Total	Male (N = 36325)	Female (N = 48810)	Total
50–59 y	17815 (42.9)	24856 (48.3)	42671 (45.9)	15062 (41.5)	23306 (47.7)	38368 (45.1)
60–69 y	14744 (35.5)	16841 (32.7)	31585 (34.0)	12694 (34.9)	15975 (32.7)	28669 (33.7)
70–79 y	6374 (15.3)	6675 (13.0)	13049 (14.0)	6035 (16.6)	6535 (13.4)	12570 (14.8)
≥80 y	2633 (6.3)	3080 (6.0)	5713 (6.1)	2534 (7.0)	2994 (6.1)	5528 (6.5)
**Literacy status**						
Illiterate	13756 (37.9)	35254 (72.2)	49010 (57.6)	13756 (37.9)	35254 (72.2)	49010 (57.6)
Upto 4^th^	8991 (24.8)	7571 (15.5)	16562 (19.5)	8991 (24.8)	7571 (15.5)	16562 (19.5)
5^th^-9^th^	7602 (20.9)	3907 (8.0)	11509 (13.5)	7602 (20.9)	3907 (8.0)	11509 (13.5)
10^th^ pass	5976 (16.5)	2078 (4.3)	8054 (9.5)	5976 (16.5)	2078 (4.3)	8054 (9.5)
**Place**						
Urban	8746 (21.0)	11829 (23.0)	20575 (22.1)	7566 (20.8)	11265 (23.1)	18831 (22.1)
Rural	32820 (79.0)	39623 (77.0)	72443 (77.9)	28759 (79.2)	37545 (76.9)	66304 (77.9)

The overall age-gender standardized prevalence of blindness in population aged ≥50 years was 1.99% (95% CI 1.94%, 2.13%) (Table 3). A rising trend in prevalence of blindness was noted with increasing age of the participants. The prevalence increased from 0.45% (95% CI: 0.4–0.5%) in 50–59 years to 4.11% (95% CI: 3.8–4.5%) in 70–79 years and 11.62% (95% CI 10.7–12.6%) in those aged ≥80 years. A higher prevalence was noted in females (2.31%, 95% CI: 2.18–2.45%) than in males (1.67%, 95% CI: 1.54–1.81%). Participants residing in rural regions had higher prevalence of blindness (2.14%, 95% CI: 1.72–2.47%) than those living in urban regions (1.80%, 95% CI: 1.52–2.15%). Prevalence of visual impairment (VI) was 26.68% (95% CI 26.57–27.17%), showing similar trends with age, gender and place of residence (Table 3), as was seen with blindness.

**Table 3:** Prevalence of blindness and visual impairment in population aged ≥50 years.

	Blind	Mild VI	Moderate VI	Severe VI	MSVI	VI	Pinhole Blind	Functional Low Vision
Variable	% (95% CI)	% (95% CI)	% (95% CI)	% (95% CI)	% (95% CI)	% (95% CI)	% (95% CI)	% (95% CI)
**Participants aged 50 years and above (N = 85135)**								
**Overall**	1.99	12.92	9.81	1.96	11.77	26.68	1.75	1.03
	(1.94, 2.13)	(12.83–13.32)	(9.69–10.12)	(1.92–2.11)	(11.68–12.15)	(26.57–27.17)	(1.71–1.89)	(0.98–1.12)
**Age Group**								
50–59	0.45	8.17	3.67	0.55	4.22	12.84	0.41	0.32
	(0.4–0.5)	(7.38–9.04)	(3.07–4.39)	(0.43–0.69)	(3.51–5.06)	(12.63–13.08)	(0.34–0.50)	(0.26–0.40)
60–69	1.58	15.13	11.15	1.85	13.00	29.71	1.34	0.59
	(1.3–1.7)	(13.95–16.39)	(9.89–12.56)	(1.48–2.29)	(11.46–14.71)	(29.37–29.99)	(1.01–1.77)	(0.46–0.76)
70–79	4.11	20.18	19.68	4.29	23.97	48.26	3.46	1.37
	(3.8–4.5)	(19.05–21.35)	(18.06–21.41)	(3.73–4.93)	(21.90–26.17)	(47.78–48.46)	(2.89–4.14)	(1.14–1.66)
≥80	11.62	21.32	26.27	8.12	34.39	67.33	10.35	2.73
	(10.7–12.6)	(19.94–22.76)	(24.25–28.41)	(7.09–9.29)	(32.05–36.81)	(66.91–67.54)	(8.76–12.18)	(2.09–3.57)
**Gender**								
Male	1.67	11.90	9.21	1.60	10.82	24.39	1.43	0.93
	(1.54–1.81)	(11.55–12.26)	(8.91–9.53)	(1.48–1.74)	(10.49–11.17)	(23.89–24.90)	(1.31–1.56)	(0.83–1.03)
Female	2.31	13.94	10.41	2.32	12.73	28.98	2.07	1.13
	(2.18–2.45)	(13.61–14.28)	(10.13–10.70)	(2.18–2.46)	(12.41–13.05)	(27.50–28.10)	(1.95–2.21)	(1.04–1.23)
**Residence**								
Rural	2.14	13.18	10.31	2.10	12.41	27.73	1.82	1.12
	(1.72–2.47)	(12.13–14.31)	(9.31–11.41)	(1.28–2.47)	(11.12–13.84)	(27.35–27.95)	(1.51–2.19)	(1.04–1.20)
Urban	1.80	12.72	8.73	1.66	10.4	24.92	1.59	1.06
	(1.52–2.15)	(11.95–13.54)	(7.90–9.64)	(1.4–1.97)	(9.42–11.46)	(24.63–25.21)	(1.34–1.88)	(0.92–1.21)

MSVI: moderate-severe visual impairment; VI: visual impairment.

**Fig 1:**
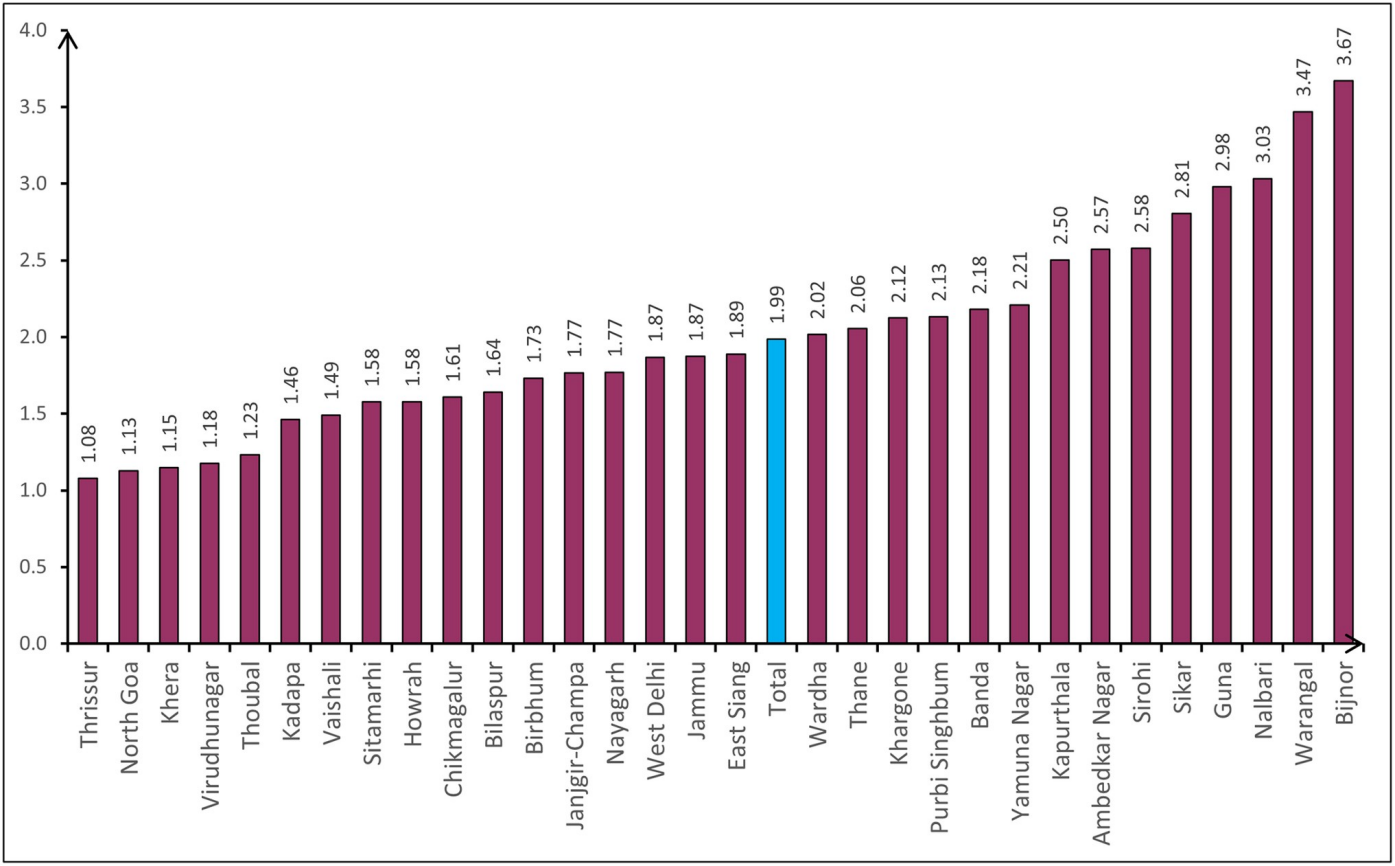
District-wise prevalence of blindness (PVA Better eye <3/60) in population aged ≥ 50 years in 31 districts of India.

Among the 31 surveyed districts, the highest prevalence was seen in Bijnor, Uttar Pradesh (3.7%: 2.9–4.4), whereas the lowest was seen in Thrissur, Kerala (1.1%: 0.6–1.5) (Fig 1). Prevalence of blindness was higher among females than males in all districts except Khera (Gujarat), Birbhum (West Bengal) and Kadapa (Andhra Pradesh) where prevalence among males was higher. In Warangal (Telangana) there was equal prevalence among males and females.

**Fig 2:**
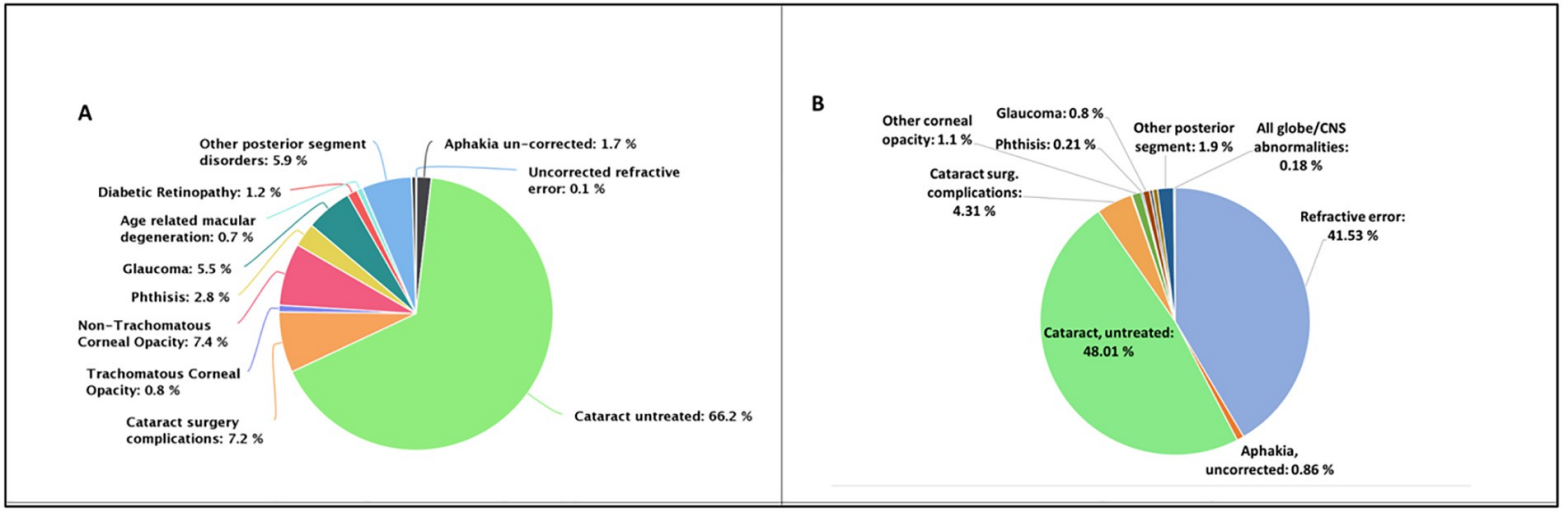
Causes of blindness and visual impairment in population aged ≥50 years (A and B respectively).

On multivariate analysis, adjusted odds ratio showed that blindness was associated with age more than 80 years (OR = 20.3, 95% CI: 15.6–26.4) and being illiterate (OR = 5.6, 95% CI: 3.6–8.9) (Table 4). Those aged 80 years and above had about twenty times higher risk of becoming blind than those aged 50–59 years (p<0.001). Participants who were illiterate had nearly six times higher odds of being blind than those who were educated (10^th^ class and above) (p<0.001). Major causes of blindness included cataract (66.2%), corneal opacity (CO) (8.2%), cataract surgical complications (7.2%), posterior segment disorders (5.9%) and glaucoma (5.5%) (Fig 2A). Untreated cataract (48.01%) followed by uncorrected refractive errors (41.53%) were the major contributors to visual impairment (Fig 2B). Out of all the individuals examined, 11.5% had uncorrected refractive error and 74.2% had uncorrected presbyopia.

**Table 4:** Multi-logistic regression analyses showing association of blindness with socio-demographic factors in population aged ≥50 years (N = 85135).

Variable		Unadjusted		Adjusted	
Age (years)	Yes (%)	OR (95% CI)	p-value	OR (95% CI)	p-value
50–59	0.21	1		1	
60–69	0.53	3.3 (2.7, 3.9)	*<0*.*001*	3.0 (2.4, 3.9)	*<0*.*001*
70–79	0.57	8.9 (7.5, 10.6)	*<0*.*001*	7.2 (6.2, 8.5)	*<0*.*001*
≥80	0.68	31.5 (26.5–37.3)	*<0*.*001*	20.3 (15.6, 26.4)	*<0*.*001*
**Gender**					
Male	0.87	1		1	
Female	1.12	1.2 (1.1, 1.3)	*0*.*006*	0.9 (0.8, 1.1)	0.281
**Education**					
≥10^th^ class	0.04	1		1	
6^th^to 9^th^ class	0.05	0.9 (0.5, 1.5)	0.644	0.9 (0.6, 1.5)	0.768
Up to 4th	0.16	1.8 (1.1, 3.1)	0.028	1.6 (0.9, 2.8)	0.075
Illiterate	1.74	8.1 (5.3, 12.3)	*<0*.*001*	5.6 (3.6, 8.9)	*<0*.*001*
**Cluster Type**					
Urban	0.51	1		1	
Rural	1.48	1.11 (1.0, 1.42)	*0*.*094*	0.83 (0.7, 1.0)	*0*.*066*

**Fig 3:**
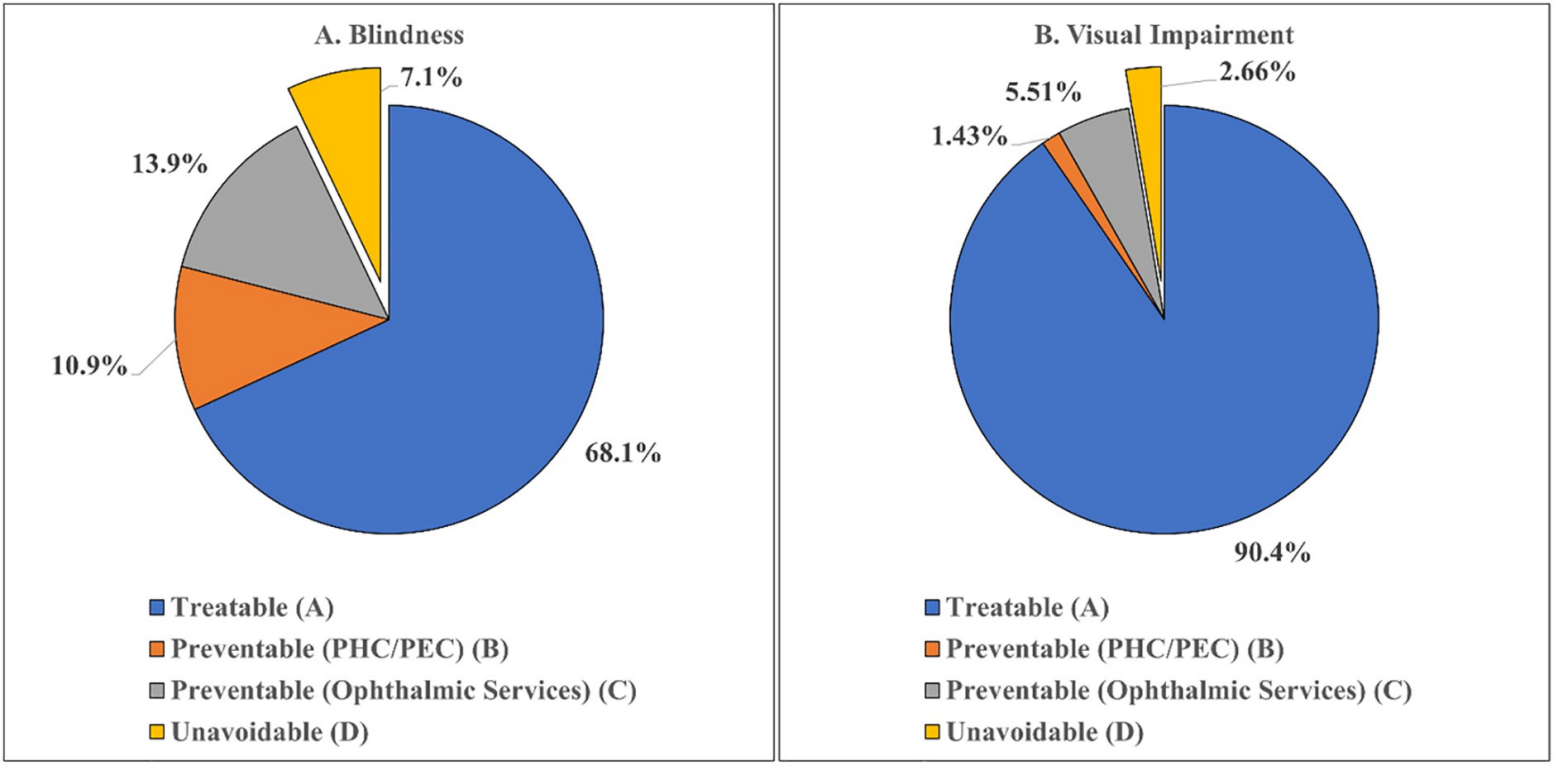
Categories of blindness and visual impairment by intervention (A and B respectively).

The proportion of avoidable blindness and avoidable visual impairment in the current study was 92.9% and 97.4% respectively. Among avoidable causes of blindness and VI, treatable causes were 68.1% and 90.4% respectively (Fig 3A and 3B).

## Discussion

The study was conducted in 31 districts of India in active partnership with ophthalmology departments of medical colleges, non-governmental organizations (NGOs), “Vision 2020: The Right to Sight” partners and Regional Institutes of Ophthalmology (RIOs), depending on the location of the clusters. Concerted efforts have been made in the last few decades to eliminate avoidable causes of blindness and visual impairment in India. Nationally representative surveys have been conducted at periodic intervals to assist NPCB&VI in assessing the impact of ongoing eye care services. The last representative survey on blindness in India was conducted in 2006–07 using RAAB protocol among individuals aged 50 years and above [9]. RAAB surveys form the key source of data on visual impairment and blindness from LMICs [18]. They use a rapid method of examination and random cluster selection, are less expensive and utilize a standardized methodology that enables valid comparisons across countries and regions [13, 19]. The RAAB surveys conducted in other South Asian countries like Thailand and Malaysia have been able to provide estimates at the country level only [20, 21]. The approach adopted in the current survey enabled us to generate both district and country level estimates. By accounting for differences in population sizes and gender structures of sampled districts, the survey results are likely to be more valid.

In the population aged 50 years and above, the prevalence of blindness has remarkably declined over past two decades from 5.3% in 2001 to 3.60% in 2007 to 1.99% in the current survey [8, 9]. Similarly, the prevalence of MSVI (PVA<6/18 to 3/60) in this group has reduced from 24.85% in 2001 to 16.8% in 2007 to 11.77% in the current survey [8, 9]. For the year 2010, Malhotra et al, in their meta-analysis, also demonstrated similar favourable trend [22]. Inequity in access to preventive and curative eye care services may explain a higher prevalence of blindness in female, elderly and illiterate participants.

Untreated cataract continues to be the most common cause of blindness and VI in adults aged 50 years or more despite nearly 6.5 million cataract surgeries conducted in India with an average cataract surgical rate of nearly 5000 per million population per year [23]. Socioeconomic development and better health care provisions in the recent years has increased the life expectancy and hence the proportion of ageing population in the country. Irrespective of socio-economic status, the non-communicable diseases (NCDs), including blindness, requiring large quantum of health and social care are extremely common in old age [24]. Corneal opacity emerged as the second most common cause of blindness in this survey. Majority of the avoidable blindness burden due to CO was attributed to non-trachomatous causes and this emphasises the need of strengthening preventive strategies and eye banking services in the country. Proportion of visual impairment due to uncorrected refractive error has reduced considerably compared to previous studies [25]. Adoption of intraocular lens implantation as a routine practice following cataract surgery has decreased visual impairment due to uncorrected aphakia but at the same time an increase in visual impairment due to cataract surgical complications was observed. Effective surgical techniques and a skilled cataract surgeon are essential for successful outcome following cataract surgery [26]. Along with increasing the coverage of eye care services, delivery of a standard quality of care needs to be ensured. Other lesser important causes of blindness included posterior segment disorders and glaucoma. These two latter causes assume greater significance in the more industrialized nations, as reported by a blindness study from Central Europe [3].

The last nationally representative data on blindness in population aged 0–49 years was collected in 1986–89 Survey of Blindness in India [8, 9]. In subsequent surveys, various assumptions were made to generate and extrapolate data to all age groups at the national level. India has a relatively younger population with about 83% of the population under 50 years of age [27]. Causes of blindness affecting this age group pose further threat as it affects the future productive force of the country. In a recent survey conducted among 0–49 years age group by NPCB&VI, Government of India, the prevalence of blindness and visual impairment reported in the 0–49 years age group was 0.52/thousand and 15.38/thousand respectively [28]. Using these proportions, the country-wide prevalence of blindness and visual impairment was estimated to be 0.36% and 5.47% respectively (Table 5). The extrapolated number of blind and visually impaired in overall population for the year 2017 was estimated to be 4.8 million and 74.0 million respectively based on population projections [29].

**Table 5:** Prevalence of blind and visually impaired in overall population of India.

Category	0–49 years	≥50 years	Total Population
	Prevalence/ 1000	Prevalence (%)	Prevalence (%)
Blindness	0.52	1.99	0.36
Severe VI	0.48	1.96	0.35
Moderate VI	3.62	9.81	1.84
Mild VI	11.05	12.92	2.92
MSVI	3.81	11.77	2.19
VI	15.38	28.68	5.47
Pinhole Blindness	0.52	1.75	0.32

MSVI: moderate-severe visual impairment; VI: visual impairment.

**Fig 4:**
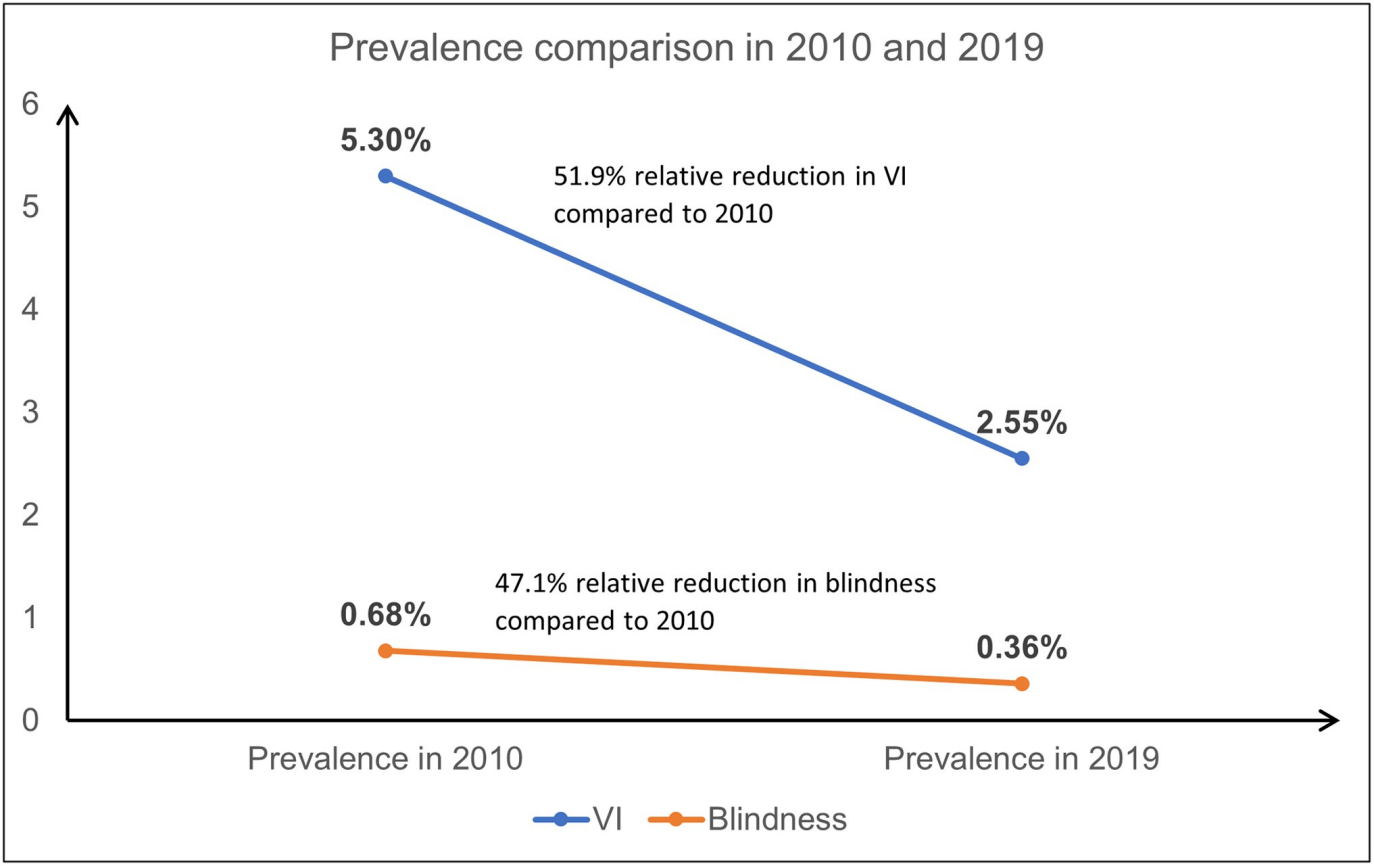
Reduction in blindness and visual impairment from 2010 estimates in overall population.

The WHO Global Action Plan for Universal Eye Health 2014–2019 targets a reduction in the prevalence of Avoidable Visual Impairment (previously defined as presenting visual acuity less than 6/18 in better eye) by 25% by the year 2019 from the baseline level of 2010. The WHO estimated a prevalence of blindness and VI as 0.68% and 5.30% respectively in India for the year 2010. The current survey shows a reduction by 47.1% in blindness and 51.9% in VI compared to the baseline levels. The target of 25% reduction in visual impairment has been successfully achieved by India (Fig 4). However, for planning and local actions, disaggregated data per district will be needed to assist in programmatic interventions and prioritization. However, it is also noted that global estimates reported that the GAP target was not met and that ageing populations and the strong association between vision impairment and age were an important barrier in reaching the target [30].

The avoidable causes of blindness include treatable causes (refractive error, aphakia uncorrected, cataract uncorrected) and preventable causes. The preventable causes include those preventable by primary health care/primary eye care (PHC/PEC) services like trachomatous corneal opacity, non-trachomatous corneal opacity, phthisis as well as those preventable by ophthalmic services like cataract surgical complications, glaucoma and diabetic retinopathy. The unavoidable causes include age related macular degeneration (ARMD), posterior segment diseases and globe/central nervous system (CNS) abnormalities. In the current study, the proportion of blindness and visual impairment due to avoidable causes was 92.9% and 97.4% respectively. According to WHO, blindness is curable or preventable in approximately 80% of cases [31]. The Global Burden Of Disease Study had reported that blindness due to cataract and under-corrected refractive error encompasses 50% of all global blindness in 2020 [30]. However, previous studies from the Indian sub-continent had reported avoidable blindness ranging from 80–95%, with wide geographical disparities [9, 32–35]. A survey on DR was also conducted in twenty-one districts out of the thirty-one covered in the current survey, in which the prevalence of DR among persons with diabetes was 16.9%, prevalence of STDR was 3.6%, and that of mild retinopathy was 11.8% [16]. This highlights the pressing need of targeting the avoidable causes in the country, which can be easily treated or prevented.

There remain several districts in India, with little or no population-based data where estimates rely on extrapolation from other regions. The findings of the current study spanning thirty-one districts out of 641 does not provide the status of blindness in the remaining districts. Only state and national level estimates can be deduced. Furthermore, RAAB is not a detailed blindness survey: it provides a reasonably accurate estimate of the prevalence of blindness, and the proportion that is avoidable in a geographic area. RAAB is not designed to give accurate estimates of the prevalence of specific diseases. The estimates for causes of blindness are biased towards more avoidable causes, especially cataract. The small numbers of people found to be blind in the survey means that extrapolations and sub-group analyses has wide confidence limits. The absence of specialised diagnostic equipment meant that cases of posterior segment disease such as glaucoma and age-related macular degeneration were identified by clinical examination only which meant that results for posterior segment causes of blindness must be interpreted with caution.

## Conclusion

The present study shows that India has made significant strides towards elimination of avoidable blindness. The prevalence of blindness and visual impairment has reduced significantly since the last national survey. However, the problem still poses a significant challenge in achieving universal eye health and needs a comprehensive and dedicated approach to effectively tackle the situation. In particular, focus is required to improve the eye banking and corneal transplantation services and cataract surgical practices in the country and to integrate refractive services effectively in the existing eye care system.

## Supporting information

S1 File (DOCX)
