# Supplementary material for: Blindness and visual impairment and their causes in India: Results of a nationally representative survey
Source: PLoS One. 2022 Jul 21;17(7):e0271736. doi: 10.1371/journal.pone.0271736 (PMC9302795; doi:10.1371/journal.pone.0271736)
Supplement: S1 File — (DOCX) [file pone.0271736.s001.docx]

Supplement 1: Clinical protocol for NB&VI Survey 2015-2019

The survey followed the standard RAAB ver. 6 protocol

Participant Enrolled, Written Informed Consent

Section A:

Demographic details and education status

Section B:

Vision

History of wearing glasses distance/near

Section E:

Reasons, if not examined

Presenting distance Visual Acuity

Section C:

Lens Examination

Section G:

Cataract Surgery Details (eye-wise), If Pseudophakia/ Aphakia

Pinhole Visual Acuity

Section D:

Main Cause of Visual Impairment

(for each eye and in person)

Section F:

Barriers to Cataract Surgery, If Cataract in any eye with pinhole VA <6/12
